# 4,4′-Bipyridine–2,3,4,5,6-penta­fluoro­benzoic acid (1/2)

**DOI:** 10.1107/S1600536809034783

**Published:** 2009-09-12

**Authors:** Xiangdong Zhang, Lijuan Wang, Chunhua Ge, Yanmei Men, Rui Zhang

**Affiliations:** a College of Chemistry, Liaoning University, Shenyang 110036, People’s Republic of China

## Abstract

In the title 1:2 adduct, C_10_H_8_N_2_·2C_7_HF_5_O_2_, the complete 4,4′-bipyridine mol­ecule is generated by a crystallographic twofold axis. The components of the adduct are linked by inter­molecular O—H⋯N hydrogen bonds and further connected by a combination of C—H⋯O, C—H⋯F and F⋯F [2.859 (2) Å] inter­actions.

## Related literature

For further discussion of inter­molecular inter­actions involving fluorine atoms, see, for example: Chopra & Row (2008 ▶); Choudhury & Row (2004 ▶).
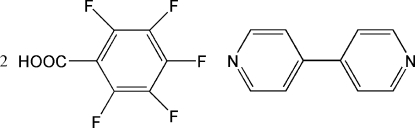


## Experimental

### 

#### Crystal data

C_10_H_8_N_2_·2C_7_HF_5_O_2_
*M* *_r_* = 580.34
Monoclinic, 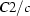

*a* = 17.910 (3) Å
*b* = 10.7016 (19) Å
*c* = 13.498 (3) Å
β = 119.631 (3)°
*V* = 2248.8 (7) Å^3^
*Z* = 4
Mo *K*α radiation
μ = 0.17 mm^−1^
*T* = 296 K
0.30 × 0.28 × 0.20 mm

#### Data collection

Bruker SMART CCD diffractometer
Absorption correction: multi-scan (*SADABS*; Bruker, 2001 ▶) *T*_min_ = 0.946, *T*_max_ = 0.974
6884 measured reflections
2695 independent reflections
2060 reflections with *I* > 2σ(*I*)
*R*_int_ = 0.022

#### Refinement

*R*[*F* ^2^ > 2σ(*F* ^2^)] = 0.041
*wR*(*F* ^2^) = 0.120
*S* = 1.05
2695 reflections
183 parameters
H-atom parameters constrained
Δρ_max_ = 0.25 e Å^−3^
Δρ_min_ = −0.18 e Å^−3^

### 

Data collection: *SMART* (Bruker, 2001 ▶); cell refinement: *SAINT* (Bruker, 2001 ▶); data reduction: *SAINT*; program(s) used to solve structure: *SHELXS97* (Sheldrick, 2008 ▶); program(s) used to refine structure: *SHELXL97* (Sheldrick, 2008 ▶); molecular graphics: *SHELXTL* (Sheldrick, 2008 ▶); software used to prepare material for publication: *SHELXL97*.

## Supplementary Material

Crystal structure: contains datablocks I, global. DOI: 10.1107/S1600536809034783/hb5082sup1.cif

Structure factors: contains datablocks I. DOI: 10.1107/S1600536809034783/hb5082Isup2.hkl

Additional supplementary materials:  crystallographic information; 3D view; checkCIF report

## Figures and Tables

**Table 1:** Hydrogen-bond geometry (Å, °)

*D*—H⋯*A*	*D*—H	H⋯*A*	*D*⋯*A*	*D*—H⋯*A*
O2—H2⋯N1	0.82	1.78	2.602 (2)	176
C9—H9⋯O1	0.93	2.40	3.102 (2)	132
C10—H10⋯O1^i^	0.93	2.35	3.196 (2)	152
C12—H12⋯F5^ii^	0.93	2.48	3.126 (2)	127
C13—H13⋯F5^ii^	0.93	2.63	3.214 (2)	121

Symmetry codes: (i) 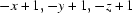; (ii) 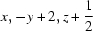.

**Fig. 1.**
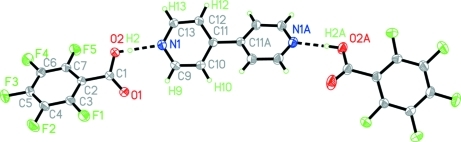
The structure of title adduct. Displacement ellipsoids are drawn at the 30% probability level. Atoms with the suffic A are generated by the symmetry operation (1–x, y, 3/2–z).

## supplementary crystallographic information

### Comment

Weak interactions involving fluorine is of great interest and importance in producing new suppramolecular assemblies. Fluorine can provide C—H···F, N—H···F hydrogen bonds (e.g. Chopra & Row, 2008) as well as C—F···F and C—F···π interactions (e.g. Choudhury & Row, 2004). in crystal engineering. Fluorine derivatives have generated a wide variety of crystal structures. The title molecular complex is composed of 4,4'-bipyridine and 2,3,4,5,6-pentafluorobenzoic acid with the molar ratio of 1:2 to form a basic unit. The components are linked by O—H···N hdrogen bond (O2···N1 2.602 (2) Å, O2—H2···N1 176 °) (Fig. 1). C9—H9···O1 weak hydrogen bond further strengthen the connection (Table 1). Intermolecular C10—H10···O1(symmery code: -*x* + 1, -*y* + 1, -*z* + 1), C12—H12···F5, C13—H13···F5 (symmery code: *x*, -*y* + 2, *z* + 1/2) hydrogen bonds and F1···F3 [2.859 (2) Å, symmery code: *x*, -*y*, -1/2 + *z*] interaction link these units further.

### Experimental

A solution of 4,4'-bipyridine (2 mmol) in ethanol (5 ml) was added into 2,3,4,5,6-pentafluorobenzoic acid (4 mmol) in ethanol(20 ml). The mixture was refluxed with stirring for 10 min. The resultant solution was filtered. Colourless blocks of (I) were formed after a few days of slow evaporation of the solvent at room temperature.

### Refinement

All H atoms were placed in calculated positions and included in a riding-model approximation, with C—H = 0.93 Å, O—H = 0.82Å and *U*_iso_(H)= 1.2Ueq(C) or 1.5Ueq(O).

### Crystal data

**Table:**

C_10_H_8_N_2_·2C_7_HF_5_O_2_	*F*(000) = 1160
*M**_r_* = 580.34	*D*_x_ = 1.714 Mg m^−^^3^
Monoclinic, *C*2/*c*	Mo *K*α radiation, λ = 0.71073 Å
Hall symbol: -C 2yc	Cell parameters from 187 reflections
*a* = 17.910 (3) Å	θ = 2.2–22.0°
*b* = 10.7016 (19) Å	µ = 0.17 mm^−^^1^
*c* = 13.498 (3) Å	*T* = 296 K
β = 119.631 (3)°	BLOCK, colorless
*V* = 2248.8 (7) Å^3^	0.30 × 0.28 × 0.20 mm
*Z* = 4	

### Data collection

**Table:**

Bruker SMART CCD diffractometer	2695 independent reflections
Radiation source: fine-focus sealed tube	2060 reflections with *I* > 2σ(*I*)
graphite	*R*_int_ = 0.022
ω scans	θ_max_ = 28.2°, θ_min_ = 2.3°
Absorption correction: multi-scan (*SADABS*; Bruker, 2001)	*h* = −23→22
*T*_min_ = 0.946, *T*_max_ = 0.974	*k* = −14→11
6884 measured reflections	*l* = −17→17

### Refinement

**Table:**

Refinement on *F*^2^	Secondary atom site location: difference Fourier map
Least-squares matrix: full	Hydrogen site location: inferred from neighbouring sites
*R*[*F*^2^ > 2σ(*F*^2^)] = 0.041	H-atom parameters constrained
*wR*(*F*^2^) = 0.120	*w* = 1/[σ^2^(*F*_o_^2^) + (0.0524*P*)^2^ + 1.397*P*] where *P* = (*F*_o_^2^ + 2*F*_c_^2^)/3
*S* = 1.05	(Δ/σ)_max_ < 0.001
2695 reflections	Δρ_max_ = 0.25 e Å^−^^3^
183 parameters	Δρ_min_ = −0.18 e Å^−^^3^
0 restraints	Extinction correction: *SHELXL97* (Sheldrick, 2008), Fc^*^=kFc[1+0.001xFc^2^λ^3^/sin(2θ)]^-1/4^
Primary atom site location: structure-invariant direct methods	Extinction coefficient: 0.0039 (7)

### Special details

**Table:**

Geometry. All e.s.d.'s (except the e.s.d. in the dihedral angle between two l.s. planes) are estimated using the full covariance matrix. The cell e.s.d.'s are taken into account individually in the estimation of e.s.d.'s in distances, angles and torsion angles; correlations between e.s.d.'s in cell parameters are only used when they are defined by crystal symmetry. An approximate (isotropic) treatment of cell e.s.d.'s is used for estimating e.s.d.'s involving l.s. planes.
Refinement. Refinement of *F*^2^ against ALL reflections. The weighted *R*-factor *wR* and goodness of fit *S* are based on *F*^2^, conventional *R*-factors *R* are based on *F*, with *F* set to zero for negative *F*^2^. The threshold expression of *F*^2^ > σ(*F*^2^) is used only for calculating *R*-factors(gt) *etc*. and is not relevant to the choice of reflections for refinement. *R*-factors based on *F*^2^ are statistically about twice as large as those based on *F*, and *R*- factors based on ALL data will be even larger.

### Fractional atomic coordinates and isotropic or equivalent isotropic displacement parameters (Å^2^)

**Table:**

	*x*	*y*	*z*	*U*_iso_*/*U*_eq_	
C10	0.49615 (12)	0.70420 (16)	0.63825 (14)	0.0565 (4)	
H10	0.5208	0.6330	0.6818	0.068*	
C12	0.44977 (12)	0.91468 (15)	0.61897 (14)	0.0541 (4)	
H12	0.4429	0.9890	0.6493	0.065*	
C13	0.42370 (13)	0.90600 (16)	0.50439 (14)	0.0578 (5)	
H13	0.3984	0.9755	0.4584	0.069*	
C9	0.46907 (12)	0.70404 (16)	0.52338 (15)	0.0574 (4)	
H9	0.4762	0.6316	0.4910	0.069*	
C11	0.48637 (10)	0.81117 (13)	0.68843 (12)	0.0430 (3)	
N1	0.43312 (9)	0.80276 (13)	0.45658 (11)	0.0516 (4)	
F1	0.31878 (9)	0.41200 (10)	0.13362 (9)	0.0756 (4)	
F5	0.38044 (8)	0.81354 (10)	0.04501 (10)	0.0727 (4)	
F2	0.24519 (9)	0.35110 (11)	−0.08528 (10)	0.0807 (4)	
O2	0.38410 (9)	0.76706 (11)	0.24230 (10)	0.0624 (4)	
H2	0.4019	0.7781	0.3105	0.094*	
F4	0.30691 (9)	0.74859 (12)	−0.17270 (10)	0.0802 (4)	
F3	0.23514 (9)	0.51811 (12)	−0.24105 (8)	0.0766 (4)	
C2	0.35217 (10)	0.61682 (14)	0.09940 (12)	0.0442 (3)	
C3	0.31759 (11)	0.49867 (15)	0.06163 (13)	0.0490 (4)	
O1	0.42833 (13)	0.57290 (14)	0.29718 (11)	0.0921 (6)	
C7	0.34739 (11)	0.69832 (14)	0.01702 (14)	0.0475 (4)	
C4	0.27894 (12)	0.46524 (15)	−0.05187 (14)	0.0529 (4)	
C5	0.27456 (11)	0.54934 (17)	−0.13111 (13)	0.0524 (4)	
C1	0.39257 (11)	0.65043 (16)	0.22420 (13)	0.0512 (4)	
C6	0.30987 (12)	0.66596 (16)	−0.09672 (14)	0.0527 (4)	

### Atomic displacement parameters (Å^2^)

**Table:**

	*U*^11^	*U*^22^	*U*^33^	*U*^12^	*U*^13^	*U*^23^
C10	0.0778 (12)	0.0382 (8)	0.0439 (9)	0.0111 (8)	0.0227 (8)	0.0021 (6)
C12	0.0787 (11)	0.0355 (8)	0.0413 (8)	0.0068 (8)	0.0244 (8)	−0.0005 (6)
C13	0.0836 (12)	0.0384 (8)	0.0409 (8)	0.0075 (8)	0.0229 (8)	0.0029 (7)
C9	0.0754 (11)	0.0435 (9)	0.0466 (9)	0.0083 (8)	0.0250 (8)	−0.0065 (7)
C11	0.0513 (8)	0.0341 (7)	0.0379 (8)	−0.0014 (6)	0.0177 (6)	−0.0004 (6)
N1	0.0633 (8)	0.0464 (8)	0.0388 (7)	0.0000 (6)	0.0205 (6)	−0.0036 (5)
F1	0.1289 (11)	0.0458 (6)	0.0587 (6)	−0.0090 (6)	0.0515 (7)	0.0036 (5)
F5	0.1063 (9)	0.0427 (6)	0.0662 (7)	−0.0179 (6)	0.0403 (7)	−0.0041 (5)
F2	0.1197 (10)	0.0498 (6)	0.0704 (7)	−0.0246 (7)	0.0454 (7)	−0.0210 (5)
O2	0.0914 (9)	0.0431 (6)	0.0387 (6)	0.0117 (6)	0.0214 (6)	−0.0040 (5)
F4	0.1189 (10)	0.0679 (8)	0.0561 (6)	0.0000 (7)	0.0450 (7)	0.0184 (6)
F3	0.1011 (9)	0.0798 (8)	0.0386 (5)	−0.0019 (7)	0.0268 (6)	−0.0116 (5)
C2	0.0529 (8)	0.0380 (7)	0.0384 (7)	0.0070 (6)	0.0200 (7)	−0.0006 (6)
C3	0.0690 (10)	0.0380 (8)	0.0429 (8)	0.0035 (7)	0.0298 (8)	0.0024 (6)
O1	0.1500 (15)	0.0554 (8)	0.0406 (7)	0.0404 (9)	0.0239 (8)	0.0032 (6)
C7	0.0571 (9)	0.0352 (7)	0.0473 (8)	0.0003 (7)	0.0236 (7)	−0.0007 (6)
C4	0.0691 (11)	0.0394 (8)	0.0505 (9)	−0.0048 (7)	0.0298 (8)	−0.0087 (7)
C5	0.0616 (10)	0.0552 (10)	0.0365 (8)	0.0046 (8)	0.0213 (7)	−0.0049 (7)
C1	0.0627 (10)	0.0439 (9)	0.0390 (8)	0.0103 (7)	0.0190 (7)	−0.0018 (6)
C6	0.0677 (10)	0.0478 (9)	0.0440 (8)	0.0054 (8)	0.0287 (8)	0.0088 (7)

### Geometric parameters (Å, °)

**Table:**

C10—C9	1.377 (2)	F2—C4	1.3383 (19)
C10—C11	1.385 (2)	O2—C1	1.295 (2)
C10—H10	0.9300	O2—H2	0.8200
C12—C13	1.380 (2)	F4—C6	1.3352 (19)
C12—C11	1.389 (2)	F3—C5	1.3328 (18)
C12—H12	0.9300	C2—C7	1.382 (2)
C13—N1	1.332 (2)	C2—C3	1.389 (2)
C13—H13	0.9300	C2—C1	1.512 (2)
C9—N1	1.330 (2)	C3—C4	1.380 (2)
C9—H9	0.9300	O1—C1	1.202 (2)
C11—C11^i^	1.483 (3)	C7—C6	1.381 (2)
F1—C3	1.3358 (18)	C4—C5	1.370 (2)
F5—C7	1.3388 (18)	C5—C6	1.372 (3)
			
C9—C10—C11	119.51 (15)	C3—C2—C1	120.42 (14)
C9—C10—H10	120.2	F1—C3—C4	116.50 (15)
C11—C10—H10	120.2	F1—C3—C2	121.41 (14)
C13—C12—C11	119.23 (15)	C4—C3—C2	122.08 (15)
C13—C12—H12	120.4	F5—C7—C6	116.66 (14)
C11—C12—H12	120.4	F5—C7—C2	120.75 (14)
N1—C13—C12	123.04 (15)	C6—C7—C2	122.59 (15)
N1—C13—H13	118.5	F2—C4—C5	119.63 (15)
C12—C13—H13	118.5	F2—C4—C3	120.38 (15)
N1—C9—C10	123.05 (16)	C5—C4—C3	119.99 (16)
N1—C9—H9	118.5	F3—C5—C4	119.92 (16)
C10—C9—H9	118.5	F3—C5—C6	120.44 (16)
C10—C11—C12	117.44 (14)	C4—C5—C6	119.63 (15)
C10—C11—C11^i^	119.81 (10)	O1—C1—O2	125.12 (15)
C12—C11—C11^i^	122.74 (10)	O1—C1—C2	121.09 (15)
C9—N1—C13	117.73 (14)	O2—C1—C2	113.78 (14)
C1—O2—H2	109.5	F4—C6—C5	120.39 (15)
C7—C2—C3	116.11 (14)	F4—C6—C7	120.03 (16)
C7—C2—C1	123.47 (15)	C5—C6—C7	119.57 (15)

Symmetry codes: (i) −*x*+1, *y*, −*z*+3/2.

### Hydrogen-bond geometry (Å, °)

**Table:**

*D*—H···*A*	*D*—H	H···*A*	*D*···*A*	*D*—H···*A*
O2—H2···N1	0.82	1.78	2.602 (2)	176
C9—H9···O1	0.93	2.40	3.102 (2)	132
C10—H10···O1^ii^	0.93	2.35	3.196 (2)	152
C12—H12···F5^iii^	0.93	2.48	3.126 (2)	127
C13—H13···F5^iii^	0.93	2.63	3.214 (2)	121

Symmetry codes:  (ii) −*x*+1, −*y*+1, −*z*+1; (iii) *x*, −*y*+2, *z*+1/2.
